# Isolation and identification of hasubanan alkaloids having anti-cholinesterase and antioxidant activity from the stem *Stephania japonica*

**DOI:** 10.1186/s12906-026-05447-7

**Published:** 2026-06-29

**Authors:** Anik Kumar Dey, Md. Yusuf Al-Amin, Rafia Ferdous, A. H. M. Khurshid Alam, Aziz Abdur Rahman, Md. Bayazid Hossen, Md. Nurul Haque Mollah, Md. Golam Sadik

**Affiliations:** 1 Department of Pharmacy, University of Rajshahi, Rajshahi, 6205 Bangladesh; 2 Department of Pharmacy, Gono Bishwabidyalay, Savar, Dhaka, 1344 Bangladesh; 3 Purdue University Interdisciplinary Life Sciences Graduate Program, Purdue University, West Lafayette, IN 47907 USA; 4 Bioinformatics Lab, Department of Statistics, University of Rajshahi, Rajshahi, 6205 Bangladesh; 5 Department of Agricultural and Applied Statistics, Bangladesh Agricultural University, Mymensingh, 2202 Bangladesh

**Keywords:** Alzheimer’s disease, *Stephania japonica*, Hasubanan, Anti-cholinesterase, Antioxidant, Molecular docking

## Abstract

**Background:**

A recent report showed that *Stephania japonica* chloroform fraction has potential anticholinesterase and antioxidant activities and is able to improve learning and memory in mice. Therefore, the aim of the present study was to isolate and identify compounds from the chloroform fraction with cholinesterase inhibitory and antioxidant activity that may be useful as new candidates for the treatment of AD.

**Methods:**

Chromatographic methods were used for isolation of compounds and the isolated compounds were analyzed by spectroscopic methods for structure elucidation. Acetyl- and butyryl-cholinesterase inhibitory activity were evaluated for by Ellman’s method and the antioxidant activity by several in vitro models such as DPPH and hydroxyl radicals scavenging, reducing power, total antioxidant activity, and inhibition of brain lipid peroxidation. The interaction of cholinesterase enzymes and isolated compounds were examined by molecular docking studies.

**Results:**

Bioactivity guided approach led to the isolation of four compounds from the chloroform fraction and identified as aknadinine, aknadilactam, aknadicine and stephisoferuline on the basis of their ^1^H-NMR and ^13^C-NMR spectral data. All the compounds were of hasubanan type. They showed significant inhibition against acetylcholinesterase and butyrylcholinesterase, with at least two fold increased affinity for butyrylcholinesterase than acetylcholinesterase. The IC_50_ values of the alkaloids were in the range of 9.36–14.89 µg/mL against acetylcholinesterase and 3.97–6.66 µg/mL against butyrylcholinesterase. Kinetic analysis revealed that all the four compounds exhibited mixed type of inhibition against both acetylcholinesterase and butyrylcholinesterase. The interaction of compounds with several amino acids of enzymes was supported by molecular docking studies. All the hasubanan alkaloids showed antioxidant activity in all in vitro assays and inhibited peroxidation of brain lipid. The IC_50_ values of the compounds for scavenging of DPPH and hydroxyl radicals, and lipid peroxidation inhibition were found to be in the range of 5.1-40.91, 10.44–19.41, and 20.60–31.72 µg/mL, respectively.

**Conclusion:**

The hasubabanan alkaloids isolated from *S. japonica* may represent a new class of anti-cholinesterase compounds. The multitargeted activity of hasubanan alkaloids may lead to new candidates for the treatment of AD.

**Supplementary Information:**

The online version contains supplementary material available at https://doi.org/10.1186/s12906-026-05447-7.

## Background

Acetylcholinesterase (AChE) is a key enzyme in cholinergic systems that plays a role in the transmission of nerve impulses. The enzyme terminates the action of the neurotransmitter acetylcholine by catalyzing its hydrolysis and regulating its level at the synapses in the brain. One of the consistent features in AD is the progressive decrease in acetylcholine, which is correlated with the loss of memory and cognition [1, 2]. Inhibitors of AChE that reduce the enzymatic degradation of acetylcholine and enhance cholinergic neurotransmission have hence been accepted as promising therapeutic approaches for treating AD. Just like AChE, butyrylcholinesterase (BChE) also hydrolyses acetylcholine but less efficiently. Nonetheless, inhibitors of BChE also increase brain acetylcholine levels and improve learning and cognitive performance in mice [3]. In the healthy human brain, AChE activity predominates over BChE activity [4]. Of the five FDA-approved drugs for AD treatment, three are cholinesterase inhibitors, namely, galantamine, donepezil and rivastigmine. All these three therapeutics inhibit AChE and, to varying extents, BChE. These drugs temporarily relieve the symptoms of AD and improve the quality of life of patients but cannot halt disease progression [5]. It is thus inferred that other pathological processes are involved in AD. Increasing evidence indicate a role for oxidative stress in the neurodegenerative process. Extensive oxidative stress has been found in the AD brain regions rich in amyloid beta (Aβ) [6]. The toxic effects of Aβ have been studied in cultured cells and animal models, which revealed that Aβ disrupts the redox balance in cells through excessive generation of free radicals, resulting in oxidative damage to proteins, lipids and DNA [7–9]. Neurons are highly susceptible to oxidative stress because of the large amount of polyunsaturated fatty acids and low level of antioxidative enzymes. Aβ-induced oxidative stress leads to a cascade of lipid peroxidation, resulting in the production of high levels of the end-products malondialdehyde (MDA) and 4-hydroxynonenal (4-HNE) in the brains of AD patients [10]. Therefore, recent approaches for AD prevention and management have focused on the use of multitargeted drugs rather than single targeted drugs [11]. Plants produce a great variety of secondary metabolites as biosynthetic intermediates that display diverse pharmacological activities [12]. A large number of phytochemicals exhibit potential for the inhibition of cholinesterase and prevention of oxidative stress [13]. The most important advantage of plant-derived medicine is its minimal side effects.

Traditional medicines, which are often used in many countries, are widely utilized as important sources of novel drugs for different diseases. Among the AD drugs approved by the FDA, galantamine was developed from the plant *Galanthus nivalis* [14]. With the goal of discovering new cholinesterase inhibitors from plants, we investigated traditional medicinal plants of Bangladesh that are used to treat neurological disorders. *Stephania japonica* (family: Menispermaceae) is a perennial herb that grows throughout Bangladesh. The plant is indicated in folk medicine for vertigo, insomnia, headache and mental disorders. Among other uses, it is used for treating cancer, urinary diseases, inflammation, fever and asthma [15, 16]. Bioactivity analysis showed that the plant possesses antidiabetic, anti-inflammatory, anticancer, analgesic and antioxidant activities. Phytochemical studies revealed a high content of alkaloids along with phenolics and flavonoids, terpenoids, steroids and saponins [17, 18]. We recently reported the anticholinesterase and antioxidant properties of this plant. Among the different fractions of the plant extract, the chloroform fraction showed the highest activity and improved learning and memory performance in mice with scopolamine-induced memory impairment [19]. However, the compounds that contribute to this activity had not been explored yet. In the present study, we aimed to isolate and identify the active compounds from the chloroform fraction of *S. japonica* and to evaluate their cholinesterase inhibitory and antioxidant activities using in vitro and molecular docking studies. Identification of these compounds could provide new drug candidates for AD.

## Materials and methods

### Chemicals

Substrates of acetylthiocholine iodide (ATCI) and S-butyrylthiocholine iodide (BTCI), 5,5′-dithio-bis-(2-nitro) benzoic acid (DTNB), 2-thiobarbituric acid (TBA) and 2-deoxy-D-ribose were procured from Sigma‒Aldrich, Germany. The reference standards donepezil, galantamine catechin, and quercetin were purchased from Sigma‒Aldrich, India. Chloroform, ethyl acetate, n-hexane, and methanol were obtained from Duksan Chemical Company, Seoul, Korea. Silica gel GF_254_, silica gel 60–120, potassium ferricyanide, and ammonium molybdate were purchased from Merck, India.

### Plant material

*Stephania japonica* stems were collected from the surrounding area (24° 22′ 0” N/88° 36′ 0” E), of Rajshahi University, Bangladesh with permission from the landowner. The plant was authenticated by the taxonomist Professor AHM Mahbubur Rahman (*collection number: 369*), and a specimen was preserved in the herbarium of the Department of Botany, Rajshahi University, Bangladesh, for reference. Following collection, they were washed with water, cut into pieces and air-dried. The dried plant parts were ground into coarse powder using a grinding machine.

### Extraction

The plant powder (1 kg) was extracted by maceration in methanol at room temperature for 7 consecutive days. The extract was filtered and concentrated by a rotary evaporator to obtain a semisolid mass (87.7 g). According to the modified Kupchan method [20], the crude methanol extract (10 g) was suspended in 180 mL water which was then partitioned with *n*-hexane for defatting and subsequently with chloroform to yield the corresponding chloroform fraction (CHF, 5.02 g). The chloroform fraction was stored at 4 °C until further experiments.

### Activity-guided isolation of compounds and identification by spectroscopic analyses

Activity-guided approach was followed for the isolation of active compounds from the chloroform fraction using chromatographic techniques. The CHF (6 g) from *S. japonica* was applied to a column (32 cm ⋅ 2.5 cm) packed with silica gel 60–120 and fractionated by sequential elution with n-hexane, chloroform, and ethyl acetate in increasing ratios to yield 200 fractions. The TLC profile of each of the fractions was examined. The fractions with similar TLC profiles were combined to yield eight major fractions MCF-1 (0.23 g), MCF-2 (0.55 g), MCF-3 (0.38 g), MCF-4 (0.37 g), MCF-5 (0.29 g), MCF-6 (0.22 g), MCF-7 (0.35 g) and MCF-8 (0.47 g) whose potential anticholinesterase activity was investigated. MCF 2, 3 and 5, which have potential activity, were chromatographed via silica gel preparative thin layer chromatography using n-hexane: chloroform: ethylacetate: methanol (3:5:1:1) as the mobile phase. Compounds 1 (45 mg) and 2 (33 mg) were purified from MCF 2, while 3 (28 mg) and 4 (30 mg) were obtained from MCF 3 and MCF 5, respectively. All the compounds were identified by ^1^H- and ^13^C-NMR spectra recorded on a Bruker DPX-400 spectrometer operating at 400 MHz. The structures of the compounds were elucidated by analysis of spectral data and by comparison with published values [21–25].

### Cholinesterase inhibition assay

Acetylcholinesterase was prepared from mouse brains, and butyrylcholinesterase was obtained from human blood as described previously [26]. The method was approved by the Institutional Animal, Medical Ethics, Biosafety and Biosecurity Committee (IAMEBBC) of the University of Rajshahi, Bangladesh (Number: 336(18)/320/IAMEBBC/IBSc). The study was conducted in compliance with ARRIVE. We followed international ethical guidelines for dealing with mice. In brief, six Swiss albino mice aged 4–8 weeks were collected from the Department of Pharmacy, Jahangirnagar University, Savar, Dhaka and sacrificed by cervical dislocation after anesthesia with sodium pentobarbital (30 mg/kg, i.p.). Immediately brain tissues were collected and washed and homogenized in Tris-buffer saline (pH 7.4) and supplemented with 1% Triton X-100 (w/v). It was then centrifuged at 10,000 rpm to obtain the crude acetylcholinesterase enzyme. The protein content in the crude enzyme was determined by the Lowry method with bovine serum albumin as a standard [27].

The widely used Ellman method was employed for the determination of cholinesterase inhibitory activity [28]. We used acetylthiocholine iodide (ATCI) as the substrate for anti-acetylcholinesterase activity assay and S-butyrylthiocholine iodide (BTCI) for anti-butyrylcholinesterase activity. The hydrolysis of the substrate by the cholinesterase enzyme acetylcholinesterase/butyrylcholinesterase was monitored with a spectrophotometer. Two hundred microliters of enzyme (0.2 U/mL) was incubated with 500 µL of compound at different concentrations (1.563, 3.125, 6.25, 12.5 and 25 µg/mL) at 37 °C for 15 min. The reaction was initiated by the addition of this solution to 3.5 mL of Ellman’s reaction mixture containing ATCI (0.5 mM) and DTNB (1 mM) in 40 mM phosphate buffer (pH 8.0), and the absorbance was measured at 412 nm in a spectrophotometer. In this assay, donepezil was used as a reference standard for acetylcholinesterase and galantamine for butyrylcholinesterase, and saline as a control. The percentage inhibition of cholinesterase activity was computed by the following formula:

$$\left[\left({\mathrm A}_{\mathrm{absorbance}\;\mathrm{of}\;\mathrm{control}}-{\mathrm A}_{\mathrm{absorbance}\;\mathrm{of}\;\mathrm{sample}}\right)/{\mathrm A}_{\mathrm{absorbance}\;\mathrm{of}\;\mathrm{control}}\right]\times100$$

### Kinetics of cholinesterase enzyme inhibition

The mode of inhibition of acetylcholinesterase and butyrylcholinesterase by the isolated compounds were determined using a fixed concentration of sample (inhibitor) at 25 µg/ml and at different concentrations (0.0875, 0.175, 0.35, 0.7 and 1.4 mM) of the substrate (ATCI/BTCI). The absorbance of the solution was monitored by a spectrophotometer at 412 nm. The experiment was carried out three times. The results were plotted in a Lineweaver‒Burk graph using the reciprocal of the substrate concentration (S^− 1^) versus the reciprocal of the reaction velocity (V^− 1^), and the intersection of the lines indicates the type of inhibition.

### Antioxidant activity assays

#### DPPH radical scavenging

The compounds 1–4 were assayed for free radical scavenging activity using DPPH radical by a previously described method [29]. Test compounds or standard catechin at different concentrations (1.569–25 µg/mL) were prepared in methanol, followed by the addition of a 0.135 mM methanolic solution of DPPH. A control solution was used that contained no sample. All the test and control solutions were kept in the dark, and after 30 min, the absorbance at 517 nm was measured with a spectrophotometer. The percent (%) scavenging activity was computed by the following equation I:

$$\%\;\mathrm{scavenging}=\left\{\left({\mathrm A}_0-{\mathrm A}_1\right)/{\mathrm A}_0\right\}\times100$$

where A_0_ is the absorbance of the control and A_1_ is the absorbance of the compound/standard.

#### Hydroxyl radical scavenging assay

The compounds 1–4 were assayed for hydroxyl free radicals scavenging activity by the deoxyribose degradation method [30]. Compounds or standard catechin at various concentrations (1.569–25 µg/mL) were prepared in 1 ml of 20 mM potassium dihydrogen phosphate buffer at pH 7.4, 2.8 mM 2-deoxy-D-ribose, 100 µM FeCl_3_, 100 µM EDTA, 1.0 mM hydrogen peroxide, and 100 µM ascorbic acid and incubated at 37 °C for 1 h. Following incubation, the mixture was added to an equal volume of 10% trichloroacetic acid (TCA) and 1% thiobarbituric acid (TBA) solution and heated for 15 min at 100 °C in a hot water bath. After cooling, the absorbance was taken at 532 nm by a spectrophotometer. A control solution was similarly prepared that contained no sample. The percent (%) scavenging activity was computed by the following equation I.

#### Ferric Reducing Power (FRP) assay

The compounds 1–4 were assayed for reducing power using a previously described method [31]. One milliliter of different concentrations of the compound or standard catechin (1.569–25 µg/mL) was mixed with equal volume of 200 mM phosphate buffer (pH 6.6) and 1% potassium ferricyanide and heated at 50 °C for 20 min, followed by cooling. Then, 10% trichloroacetic acid (TCA) was added to the mixture and centrifuged at 4000 rpm for 15 min to obtain the supernatant. The supernatant was mixed with an equal volume of double distilled water and 0.1% ferric chloride solution. The resulting solution was incubated at 37 °C for 10 min, and the absorbance of the Perl’s Prussian blue-colored solution was measured at 700 nm by a spectrophotometer. A control solution that contained no sample was used.

#### Total antioxidant activity assay

The compounds 1–4 were assayed for total antioxidant activity using a previously described method [32]. Compounds or standard ascorbic acid at different concentrations (1.569–25 µg/mL) were mixed with 28 mM sodium phosphate, 0.6 M sulfuric acid and 4 mM ammonium molybdate and heated at 95 °C for one and a half hours. The absorbance of the resulting solution was measured at 695 nm.

#### Lipid peroxidation inhibition assay

Compounds 1–4 were assayed for inhibitory activity against lipid peroxidation by a method described earlier [33]. We prepared brain lipids from mouse brain homogenates as described previously [34]. Compounds or standard catechin at various concentrations (6.25–100 µg/mL) were added to 500 µL of brain lipids and 200 µL of 0.2 mM FeCl_3_, mixed well and incubated at 37 °C for 30 min. Following incubation, the mixture was added to 2 ml of a solution of hydrochloric acid (0.25 N) containing trichloroacetic acid (15%), tert-butyl alcohol (0.38%), and butylated hydroxytoluene (5%) and heated at 80 °C for 1 h. The mixture was then centrifuged at 3000 rpm for 10 min to obtain the supernatant, and the absorbance was measured at 532 nm with a spectrophotometer. A control solution that contained no sample was used. The percent (%) inhibitory activity was computed by the following equation I.

### Molecular docking analysis of the isolated compounds with enzyme targets

To support our experimental results computationally, we performed molecular docking analysis of two enzyme proteins (acetylcholinesterase and butyrylcholinesterase) with six drug molecules, including four isolated compounds (Aknadilactam, Aknadinine, Stephisoferuline and Aknadicine) and two already approved molecules (Donepezil and Galantamine). The structures of acetylcholinesterase (ID: 4BDT, resolution: 3.10 Å) and butyrylcholinesterase (ID: 4AQD, resolution: 2.50 Å) proteins were downloaded from the online Protein Data Bank (PDB) database [35]. We selected these human proteins due to similarity in active site with that of mouse. Afterward, the Discovery Studio Visualizer 2021, Swiss PDB viewer [36] and AutoDock tools [37] were used to prepare the receptor proteins. The preparation involved the removal of water molecules, the excess copies of the enzyme chains, the addition of nonbonded inhibitors, the minimization of energy, the addition of Kollman charges, polar hydrogens and the merging of nonpolar ones. Default Gasteiger charges were assigned to all atoms. Finally, a grid box was set for each protein to define the binding site for ligand docking. The Grid box details given below in the Table S1. The 3D structures of the ligands were selected from the PubChem database and processed by setting a torsion tree through the AutoDock Tools program [37]. Finally, using the AutoDock Vina [38, 39] program, molecular docking was performed, and the binding affinity scores (BASs) between the experimental proteins (enzymes) and candidate drug agents were calculated.

### Ethical statement

Human blood was only used as the source of butyrylcholinesterase enzyme which was voluntarily donated by the researcher Anik Kumar Dey (first author). According to the guidelines set by the Institutional Animal, Medical Ethics, Biosafety and Biosecurity Committee (IAMEBBC) of the University of Rajshahi, Bangladesh, no ethical approval was required for the use of self-donated blood. Research involving minimal risk is exempt from the ethical review process by IAMEBBC.

### Statistical analysis

The data were statistically analyzed using the Statistical Package for Social Sciences (SPSS) program and Microsoft Excel 2016 software. All experiments were replicated in triplicate. The means ± SDs were determined for each experiment. The differences between the means were computed by using one-way ANOVA. *p* < 0.05 was considered as significant. GraphPad Prism 8.0.1 was used to calculate the IC_50_ values of the compounds.

## Results

### Isolation and structure elucidation of active compounds

**Fig. 1:**
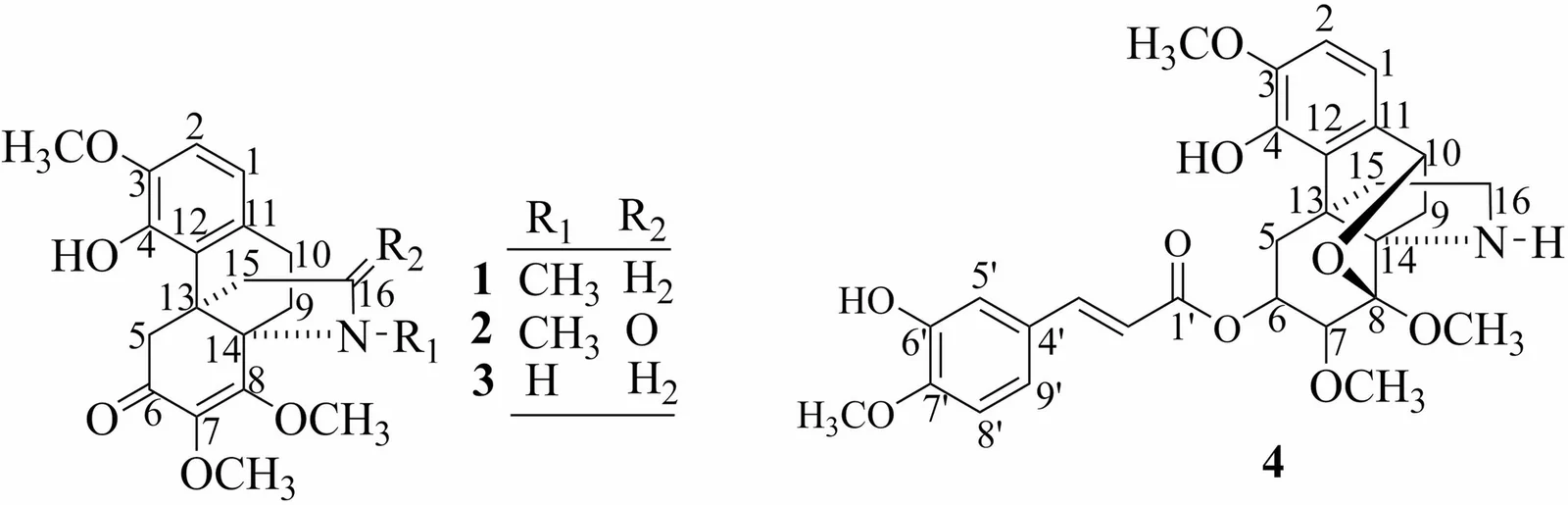
Chemical structures of the isolated hasubanan alkaloids: 1, aknadinine; 2, aknadilactam; 3, aknadicine; and 4, stephisoferuline

Bioactivity-guided fractionation was used to isolate the active compounds. The chloroform fraction, obtained by partitioning of the crude methanol extract, was chromatographed successively with column chromatography and preparative thin layer chromatography, which yielded four purified compounds, 1–4. The identification of the compounds was accomplished by ^1^H- and ^13^C-NMR spectral evidences (Tables 1, 2 and 3, Fig. S1-S8 ) and comparison with the published values [21–25]. All the compounds were found to be hasubanan alkaloids: aknadinine (1), aknadilactam (2), aknadicine (3) and stephisoferuline (4) (Fig. 1). The compounds were studied in detail for anticholinesterase and antioxidant activities in vitro as well as molecular docking.

**Table 1:** ^1^H NMR (400 MHz, CDCl3) spectroscopic data of compounds aknadinine (1), aknadilactam (2), aknadicine (3) (δ in ppm, J in Hz)^a^ and authentic aknadinine, aknadilactam and aknadicine

Position	Aknadinine (1)	Authentic aknadinine^21–23^	Aknadilactam (2)	Authentic aknadilactam^21–23^	Aknadicine (3)	Authentic Aknadicine^21–23^
1	6.56 (d, 8.4)	6.56 (d, 8.2)	6.57 (d, 8.0)	6.57 (d, 8.2)	6.59 (d, 8.3)	6.59 (d, 8.2)
2	6.66 (d, 8.0)	6.66 (d, 8.2)	6.70 (d, 8.0)	6.72 (d, 8.2)	6.69 (d, 8.3)	6.69 (d, 8.2)
3	-	-	-	-	-	-
4	-	-	-	-	-	-
5	2.64 (d, 16.0) 3.50 (d, 16.0)	2.64 (d, 16.0) 3.50 (d, 16.0)	2.79 (d, 16.0), 3.49 (d, 16.0)	2.79 (d, 16.8) 3.48 (d, 16.8)	2.52 (d, 16.7) 3.62 (d, 16.7)	2.50 (d, 16.8) 3.63 (d, 16.8)
6	-	-	-	-	-	-
7	-	-	-	-	-	-
8	-	-	-	-	-	-
9	1.93 (m) 2.14 (m)	1.90 (ddd, 13.4, 11.3, 4.6) 2.15 (ddd, 13.4, 4.9, 4.9)	2.19 (m) 2.32 (m)	2.17 (ddd, 14.1, 11.0, 5.4) 2.32 (ddd, 14.1, 5.1, 4.9)	1.86 (m) 2.14 (m)	1.82 (ddd, 13.4, 13.1, 5.2) 2.14 (ddd, 13.4, 4.9, 1.8)
10	2.58 (m) 2.78 (m)	2.56 (ddd, 16.2, 4.9, 4.6) 2.79 (ddd, 16.2, 11.3, 4.9)	2.63 (m) 2.72 (m)	2.64 (ddd, 16.8, 11.0, 4.9) 2.71 (ddd, 16.8, 5.1, 5.1)	2.62 (m) 3.08 (m)	2.61 (ddd, 17.0, 4.9, 1.8) 3.06 (ddd, 17.0, 13.1, 5.2)
11	-	-	-	-		
12	-	-	-	-		
13	-	-	-	-		
14	-	-	-	-		
15	2.14 (m) 2.47 (m)	2.11 (ddd, 14.0, 9.5, 4.0) 2.47 (ddd, 14.0, 10.1, 6.4)	2.76* 3.04*	2.76 (d, 17.1) 3.04 (d, 17.1)	2.16 (m) 2.66 (m)	2.17 (m) 2.64 (m)
16	2.68 (m) 2.84 (m)	2.67 (ddd, 9.7, 9.5, 6.4) 2.83 (ddd, 10.1, 9.7, 4.0)	-	-	2.85 (m*)	2.84 (m) 2.86 (m)
3-OMe	3.83 (s)	3.83 (s)	3.84 (s)	3.85 (s)	3.84 (s)	3.85 (s)
7-OMe	3.64 (s)	3.65 (s)	3.67 (s)	3.69 (s)	3.67 (s)	3.69 (s)
8-OMe	4.06 (s)	4.07 (s)	4.10 (s)	4.11 (s)	4.11 (s)	4.13 (s)
N-Me	2.52 (s)	2.53 (s)	2.95 (s)	2.96 (s)	-	-

*Due to overlapping signals, *J* values were not possible to determine; 21–23, references

**Table 2:** ^13^C NMR (100 MHz, CDCl3) data of compounds aknadinine (1), aknadilactam (2), aknadicine (3) (δ in ppm) and authentic aknadinine, aknadilactam and aknadicine

Position	Aknadinine (1)	Authentic aknadinine^21–23^	Aknadilactam (2)	Authentic aknadilactam^21–23^	Aknadicine (3)	Authentic aknadicine^21–23^
1	119.17	119.13	119.45	119.36	119.51	119.48
2	108.66	108.64	109.70	109.66	108.86	108.82
3	145.02	145.01	145.28	145.24	145.01	144.99
4	143.77	143.76	143.94	143.90	143.71	143.69
5	43.29	43.28	41.60	41.52	42.89	42.86
6	194.83	194.82	192.92	192.86	194.39	194.42
7	138.17	138.12	137.24	137.15	136.97	136.92
8	165.13	165.16	160.80	160.76	164.83	164.96
9	23.07	23.05	25.09	25.01	26.63	26.63
10	25.25	25.22	25.14	25.06	24.99	24.96
11	128.80	128.77	127.91	127.83	128.29	128.23
12	128.41	128.41	123.96	123.90	128.29	128.12
13	47.17	47.13	42.77	42.70	45.42	45.36
14	67.87	67.81	67.98	67.90	67.04	66.97
15	33.99	33.96	40.56	40.47	34.55	34.52
16	51.38	51.34	174.49	174.42	42.23	42.21
3-OMe	56.24	56.22	56.29	56.22	56.25	56.22
7-OMe	60.75	60.73	60.72	60.64	60.61	60.61
8-OMe	60.57	60.55	61.01	60.95	61.26	61.25
N-Me	36.38	36.36	28.23	28.16	-	-

21–23, references

**Table 3:** ^1^H (400 MHz) and ^13^C NMR (100 MHz) data of compound stehisoferulin 4 (δ in ppm, J in Hz)^a^ and authentic stephisoferulin

Position	^13^C-NMR		^1^H-NMR	
	Stephisoferuline (4)	Authentic stephisoferuline^25^	Stephisoferuline (4)	Authentic Stephisoferuline^25^
1	116.48	116.6	6.70 (d, 8.0)	6.67 (d, 8.6)
2	107.22	106.6	6.46 (d, 8.0)	6.44 (d, 8.6)
3	147.31	147.0	-	-
4	143.62	143.7	-	-
5	30.54	31.2	2.08 (m) 3.33 (m)	2.12 (dd, 15.0, 2.4) 3.14 (dd, 15.0, 3.6)
6	67.10	67.6	5.41 (m)	5.39 (m)
7	80.55	80.5	3.89 (overlapped)	3.78 (d, 3.6)
8	101.37	101.7	-	-
9	36.94	36.6	2.72 (m) 1.99 (d, 10.3)	2.68 (dd, 6.6,10.8) 1.52 (d, 10.8)
10	77.67	77.5	4.97 (d, 5.7)	4.96 (d, 6.6)
11	133.20	133.8	-	-
12	128.37	127.5	-	-
13	46.65	46.3	-	-
14	77.23	77.5	-	-
15	35.67	34.2	1.98* 2.71*	1.86 (ddd, 7.8, 10.8, 13.2) 2.50 (ddd, 3.6, 10.8, 12.6)
16	41.46	41.5	3.40 (m)	3.39 (m)
C-1ʹ	166.95	167.2	-	-
C-2ʹ	117.06	116.9	5.30 (d, 16.0)	5.33 (d, 16.2)
C-3ʹ	142.87	142.5	7.10 (d, 16.0)	7.06 (d, 16.2)
C-4ʹ	128.37	128.4	-	-
C-5ʹ	113.07	113.2	6.90 (d, 2.0)	6.91 (d, 1.8)
C-6ʹ	145.61	145.60	-	-
C-7ʹ	148.15	148.1	-	-
C-8ʹ	110.43	110.3	6.81 (d, 8.3)	6.81 (d, 8.4)
C-9ʹ	121.63	121.5	6.86 (dd, 8.3, 2.0)	6.86 (dd, 8.4, 1.8)
3-OMe	55.54	55.2	3.35 (s)	3.35 (s)
7-OMe	56.99	57.2	3.40 (s)	3.41 (s)
8-OMe	52.19	51.4	3.60 (s)	3.57 (s)
7ʹ-OMe	55.97	55.9	3.91 (s)	3.94 (s)

*Due to overlapping signals, *J* values were not possible to determine; 25, reference

### Anticholinesterase activity of the isolated compounds 1–4

**Fig. 2:**
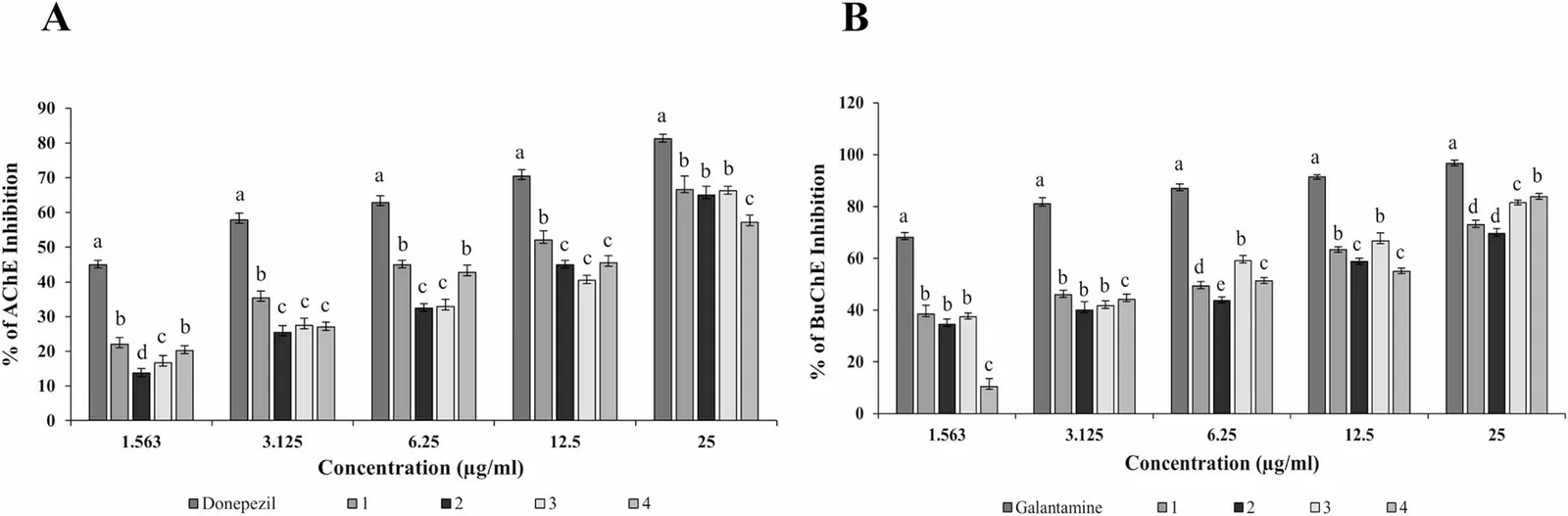
Anticholinesterase activity of compounds 1-4 isolated from *S. japonica*. **A** AChE inhibitory activity. Donepezil was used as the reference drug. **B** BChE inhibitory activity. Galantamine was used as the reference drug. The results are indicated as mean ± SD (*n* = 3). Means with different letters (a–f) differ significantly (*P* < 0.05). AChE, acetylcholinesterase; BChE, butyrylcholinesterase. 1, aknadinine; 2, aknadilactam; 3, aknadicine; 4, stephisoferuline

The anticholinesterase potential of compounds 1–4 was assessed by the Ellman method. All the compounds inhibited acetylcholinesterase in a dose-dependent manner (Fig. 2A, Table 4). For comparison of activity, the IC_50_ values (the concentration required to reduce enzymatic activity by 50%) of the compounds were determined. Compound 1 was found to be the most potent against acetylcholinesterase that gave an IC_50_ value of 9.36 ± 1.00 µg/mL. The IC_50_ values of the compounds 2, 3 and 4 were 13.84 ± 0.92, 14.37 ± 0.78 and 14.89 ± 1.24 µg/mL, respectively.

**Table 4:** IC_50_ values of the isolated compounds 1-4 in acetylcholinesterase (AChE) and butyryl-cholinesterase (BChE) inhibitory activity

Compounds	AChE Inhibitory Activity, IC50 (µg/mL)	BuChE Inhibitory Activity, IC50 (µg/mL)
1	9.36 ± 1.00	4.5 ± 0.53
2	13.84 ± 0.92	6.66 ± 0.33
3	14.37 ± 0.78	3.97 ± 0.38
4	14.89 ± 1.24	6.51 ± 0.18
Standard	2.1 ± 0.14	0.61 ± 0.09

Similar to acetylcholinesterase, the compounds also inhibited butyrylcholinesterase in a dose-dependent manner (Fig. 2B, Table 4). Compound 3 had the highest inhibitory activity against butyrylcholinesterase followed by compound 1. Their IC_50_ values were 3.97 ± 0.38 µg/mL and 4.50 ± 0.53 µg/mL, respectively. The activities of 4 and 2 were nearly equal. Comparison of the anti- acetyl- and butyrylcholinesterase activities of the compounds revealed that they show 2-3.6 times greater specificity for butyrylcholinesterase than for acetylcholinesterase.

### Determination of enzyme inhibition kinetics

**Fig. 3:**
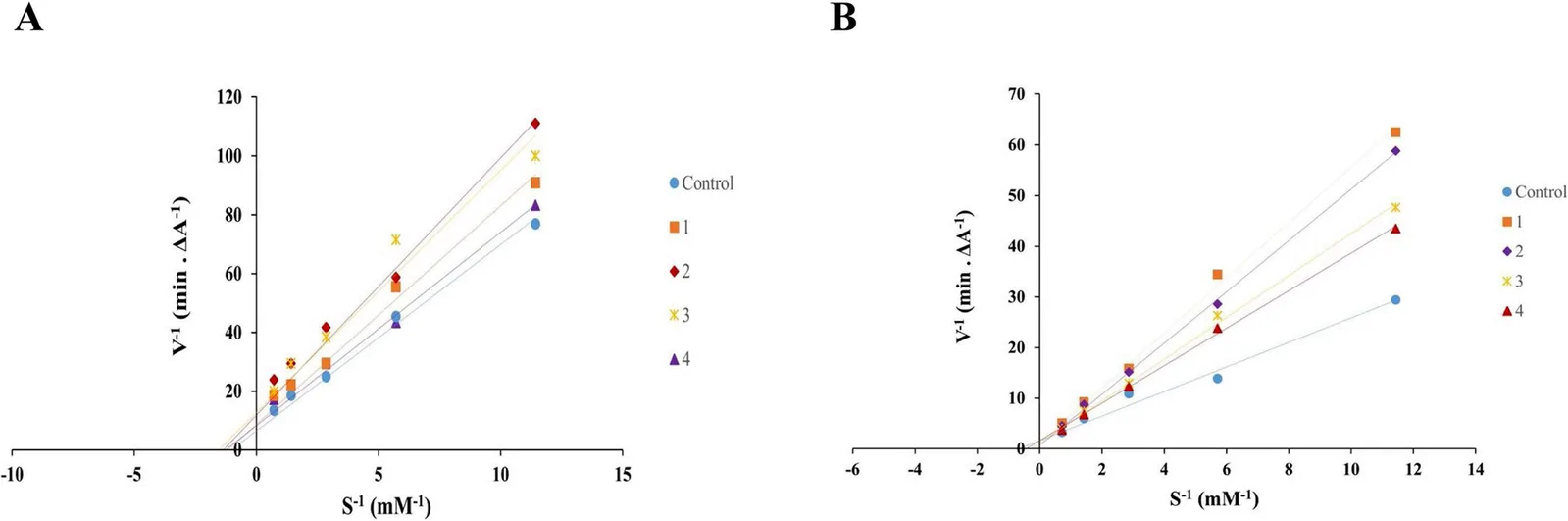
Lineweaver–Burk plot for the compounds 1-4 against AChE (**A**) and BChE (**B**). The results indicate the average values (*n*=3). 1, aknadinine; 2, aknadilactam; 3, aknadicine; 4, stephisoferuline; Control, without compound

The mode of acetyl- and butyrylcholinesterase enzyme inhibition by the isolated compounds 1–4 were determined by a double reciprocal Lineweaver‒Burk plot, and the results are shown in Fig. 3 and Table. S2. Plots of acetylcholinesterase and butyrylcholinesterase inhibition by the compounds were linear and intersected at a point neither on X-axis nor on Y-axis. The binding of the compounds to acetylcholinesterase and butyrylcholinesterase affected the K_m_ and V_max_ values, a pattern indicating that the compounds exhibited mixed type of inhibition. These results suggest that the compounds bind to an active site and a non-catalytic site in acetylcholinsterase and butyrylcholinesterase.

### Molecular docking analysis

**Fig. 4:**
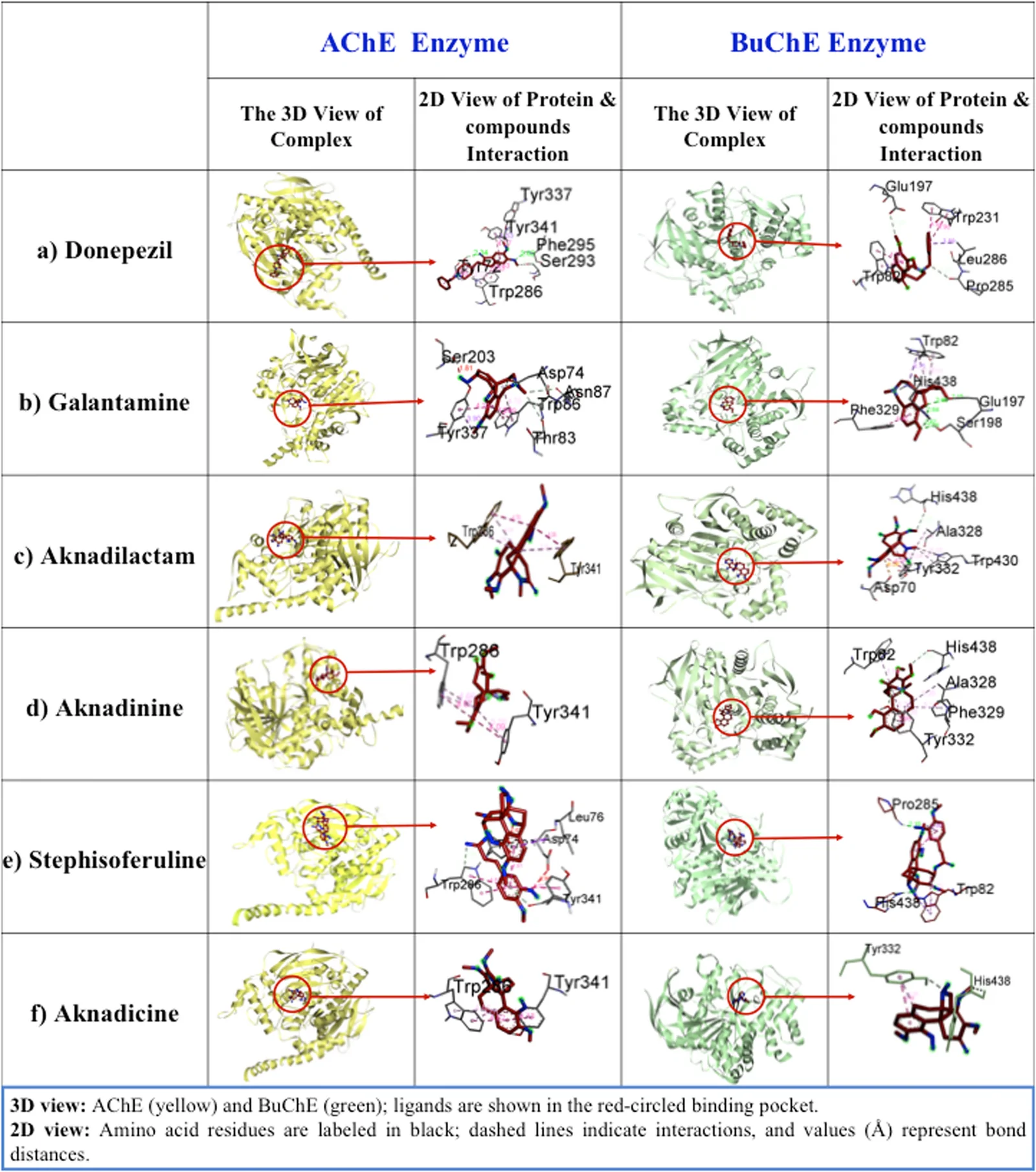
Molecular docking analysis of compounds (a–f) with AChE and BuChE enzymes. In the 3D complex view, AChE and BuChE proteins are shown in yellow and green, respectively, while the ligands are highlighted within the red circle indicating the binding pocket. In the 2D interaction view, amino acid residues involved in ligand binding are labeled in black text. Green dashed lines represent hydrogen bonds, while pink/purple dashed lines represent hydrophobic interactions, and the numerical values (Å) indicate the distances between interacting atoms

The interactions of the cholinesterase enzymes acetylcholinesterase and butyrylcholinesterase with compounds 1–4 were studied by molecular docking experiments to determine the binding affinity and the sites of interaction. Our results confirmed that all of the compounds significantly bind with the enzymes (BAS <-7.0 kcal/mol). The binding affinities of the compounds ranged from − 7.7 to -8.5 kcal/mole for acetylcholinesterase and from − 8.6 to -9.3 kcal/mol for butyrylcholinesterase (Table 5). The binding of compounds 1–4 with acetylcholinesterase involved the hydrophobic interactions at TRP286 and TYR354, indicating that they exhibit the same mechanism of enzyme inhibition (Fig. 4A). Compound 4 showed additional hydrophobic interactions with LEU76, VAL294 and TYR341, which may account for its increased affinity. The interaction of compound 1 with BChE involved H-bonding at TYR332 and HIS438 and hydrophobic interactions at ALA328, TRP82 and PHE329 (Fig. 4B). Compound 2 interacted with butyrylcholinesterase through hydrogen bonding at TYR332 and HIS438. Compound 3 interacted with butyrylcholinesterase through hydrogen bonding at HIS438 and hydrophobic interactions at TYR332, ALA328 and TRP430. Compound 4 binds through H-bonding at HIS438 and PRO285 and hydrophobic interactions at TRP82, ALA328, PHE329 and TYR332.

**Table 5:** Molecular docking of compounds aknadinine (1), aknadilactam (2), aknadicine (3) and stehisoferulin 4 with acetyl- and butyryl-cholinesterase

Proteins	Ligands	Binding Affinity (kCal/mol)	Interacting Amino Acids	
			Hydrogen Bond	Hydrophobic Interactions
	Donepezil	-8.5	TYR72, PHE295, SER293, TYR337	TRP286, TYR341
	Aknadinine	-8.3	-	TRP286, TYR341
AChE	Aknadicine	-8	-	TRP286, TYR341
	Aknadilactam	-8.5	-	TRP286, TYR341
	Stephisoferuline	-7.7	TRP286, TYR341	LEU76, TRP286, LEU76, VAL294
	Galantamine	-8.8	SER198, HIS438, GLU197, TRP82	PHE329
	Aknadinine	-8.9	HIS438, TYR332	ALA328, TRP82, PHE329
BChE	Aknadicine	-8.6	HIS438, TYR332	-
	Aknadilactam	-9.3	HIS438	TRP430, ALA328, TYR332
	Stephisoferuline	-9.3	HIS438, PRO285	TRP82, ALA328, PHE329, TYR332

‘-’ no bonds

### Antioxidant activity

#### DPPH and hydroxyl radical scavenging activity

**Fig. 5:**
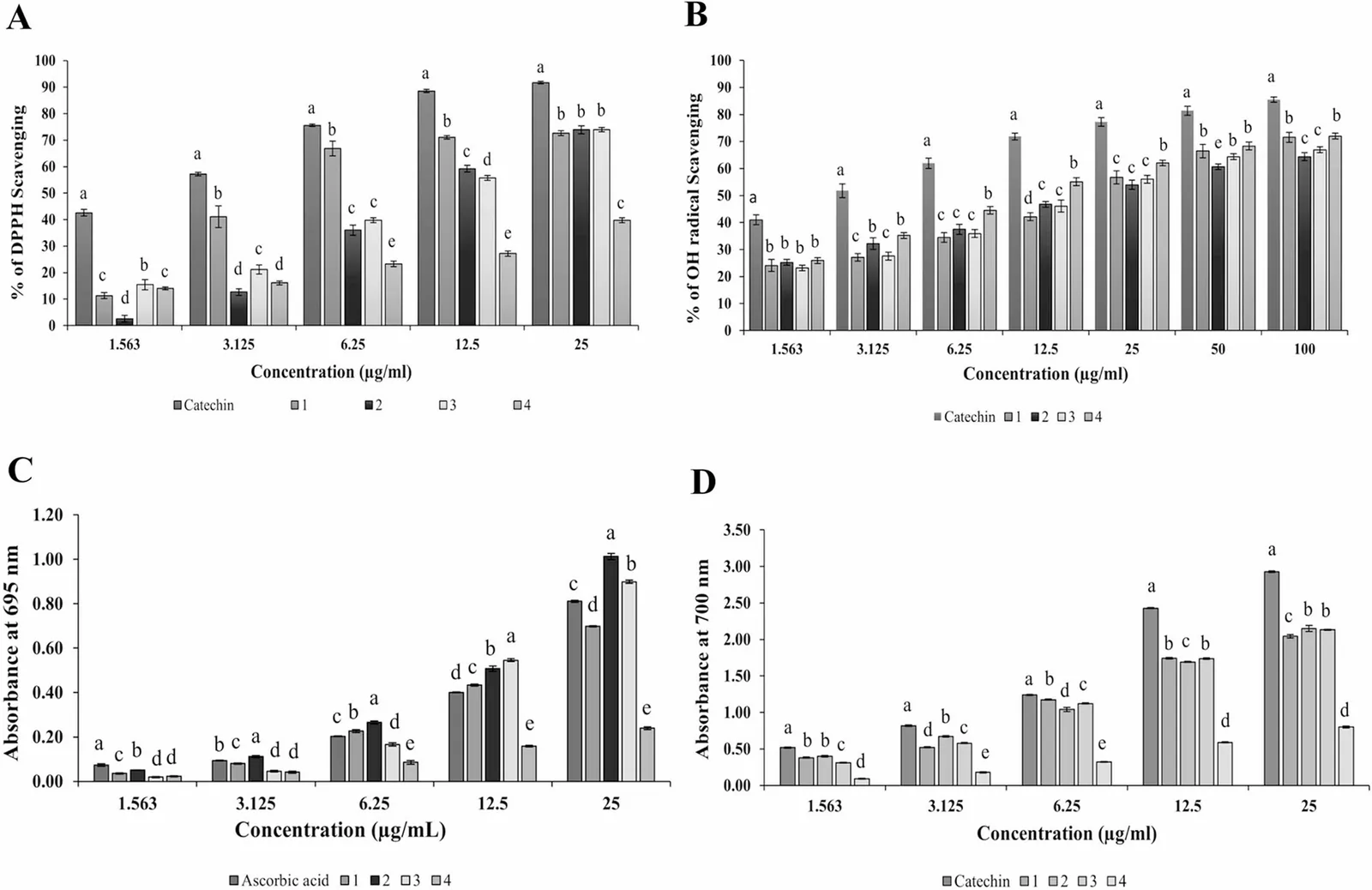
Antioxidant activities of compounds 1-4: **A** DPPH radical scavenging activity. Catechin was used as the standard. **B** Hydroxyl radical scavenging activity. Catechin was used as the standard. **C** Total antioxidant capacity. Ascorbic acid was used as the standard. **D** Ferric reducing power. Catechin was used as the standard. The results are shown as the mean ± SD (*n* = 3). In each concentration, different letters (a–f) indicate significant (*P* < 0.05) differences. 1, aknadinine; 2, aknadilactam; 3, aknadicine; 4, stephisoferuline

The free radical scavenging activity of purified compounds 1–4 was evaluated against DPPH and hydroxyl free radicals. DPPH is a synthetic free radical, and the DPPH-based assay is sensitive and reliable for assessing antioxidant activity in vitro. The percentage of scavenging by different concentrations of the compounds is presented in Fig. 5A and Table 6. All of the compounds scavenged DPPH radicals in a dose-dependent manner. The highest activity was shown by compound 1, which gave an IC_50_ value of 5.10 ± 0.42 µg/mL. Compounds 3 and 2 also showed good activity with IC_50_ values of 9.70 ± 0.44 and 10.38 ± 0.25 µg/mL, respectively.

**Table 6:** Overview of the antioxidant activity of the isolated compounds 1-4

Compounds	DPPH radical scavenging activity (IC_50_ µg/mL)	Hydroxyl radical scavenging activity (IC_50_, µg/mL)	Lipid peroxidation inhibition (IC_50_, µg/mL)	Ferric-reducing power (absorbance at 25 µg/mL)	Total antioxidant capacity (absorbance at 25 µg/mL)
1	5.10 ± 0.42	17.68 ± 2.75	31.71 ± 1.21	2.043 ± 0.025	0.698 ± 0.003
2	10.38 ± 0.25	19.41 ± 2.32	26.97 ± 1.05	2.149 ± 0.043	1.012 ± 0.014
3	9.70 ± 0.44	18.33 ± 2.07	22.60 ± 1.19	2.132 ± 0.008	0.899 ± 0.008
4	40.91 ± 0.66	10.44 ± 0.74	27.81 ± 1.98	0.800 ± 0.011	0.239 ± 0.007
Standard	2.16 ± 0.08	2.73 ± 0.41	16.17 ± 0.33	2.929 ± 0.010	1.037 ± 0.004

Hydroxyl radicals are normally produced in stressed cells in AD. We quantified the ability of compounds 1–4 to scavenge the hydroxyl radicals generated in a Fenton reaction. The percentage of free hydroxyl radicals scavenged by each compound was calculated and is presented in Fig. 5B and Table 6. Like DPPH radicals, all of the compounds scavenged hydroxyl radicals, and the activity was increased with increasing dose. Compound 4 showed the highest activity, with an IC_50_ value of 10.44 ± 0.74 µg/mL, followed by compounds 1, 3 and 2, with IC_50_ values of 17.68 ± 2.75, 18.33 ± 2.07 and 19.41 ± 2.32 µg/mL, respectively. The antioxidant potential of the hasubanan alkaloids suggests that they might be effective in preventing free radical-induced oxidative damage to cells and tissues.

#### Total antioxidant activity and reducing power of the isolated compounds

Total antioxidant activity is also a measure of the antioxidant potential of a compound. We determined the total antioxidant activity of compounds 1–4 based on the reduction of Mo(VI) to Mo(V). All of the compounds showed significant activity, and the activity of the compounds increased with increasing concentration (Fig. 5C, Table 6). Compounds 2 and 3 showed strong activity which were even stronger than the standard ascorbic acid. At 25 µg/mL, the absorbances of compounds 1–4 were 0.698 ± 0.003, 1.012 ± 0.014, 0.899 ± 0.008, and 0.239 ± 0.007, respectively.

The reductive power of compounds 1–4 was determined by their ability to reduce Fe^+ 3^ to Fe^+ 2,^ and the results are shown in Fig. 5D and Table 6. It is clear from the results that all of the compounds have significant reducing activity. Among the compounds, the highest activity was shown by compound 2, followed by compounds 1, 3 and 4. At 25 µg/mL, the absorbances of compounds 1–4 were 2.043 ± 0.025, 2.149 ± 0.043, 2.132 ± 0.008, and 0.800 ± 0.011, respectively.

#### Lipid peroxidation inhibitory activity

**Fig. 6:**
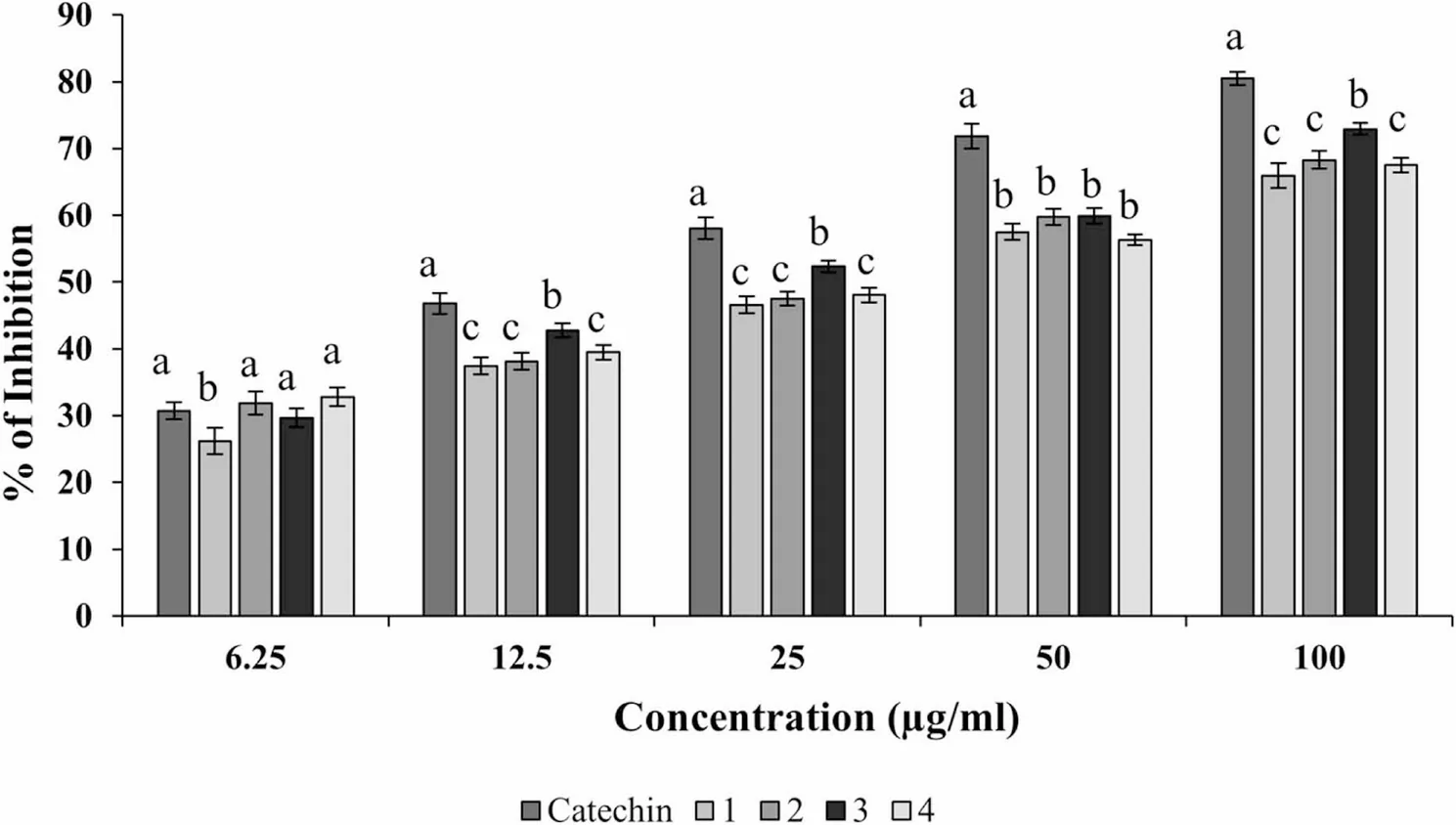
Lipid peroxidation inhibitory activity of compounds 1-4. Catechin was used as the standard. The results are shown as the mean ± SD (*n* = 3). In each concentration, different letters (a–f) indicate significant (*P* < 0.05) differences.. 1, aknadinine; 2, aknadilactam; 3, aknadicine; 4, stephisoferuline

The peroxidation of lipids results from the oxidation of lipids by free radicals, which is extensive in AD. The potential of compounds 1–4 for the inhibition of lipid peroxidation induced by hydroxyl radicals was assessed by the TBARS method. The results (Fig. 6, Table 6) were clear reflection of the antioxidant activity of the compounds. Compound 3 showed the most potent activity and its IC_50_ value was found to be 20.60 ± 1.19 µg/mL. The compounds 2, 4 and 1 showed the IC_50_ values of 26.97 ± 1.05, 27.81 ± 1.98 and 31.72 ± 1.21 µg/mL, respectively. The findings clearly indicate that all of the compounds are able to prevent the peroxidation of lipids through the scavenging of free radicals.

## Discussion

AD is a chronic neurodegenerative disease that mostly affects the elderly population. As age advances, so does the incidence of AD. At an age above 80, 30% of people suffer from dementia, which poses a serious threat to society [40]. The number of deaths from cardiovascular disease decreased between 2000 and 2019, but the number of deaths from AD increased significantly [41]. These data demand novel treatments for the management of this disease. Recently, traditional medicine has received much attention as a source of drugs because they contain diverse chemical compounds with potential pharmacological activities [42]. Moreover, many are considered to be safe and are affordable. *S. japonica* is a folk medicine used by the rural people in Bangladesh to treat different neurological disorders and ailments [15, 16]. In a previous study, we showed that the chloroform fraction of this plant extract has potential neuroprotective effects and improves learning and memory performance in mice through inhibition of cholinesterase and prevention of oxidative stress [19]. In this study, we report the isolation of hasubanan alkaloids from *S. japonica* with strong cholinesterase inhibitory activity and antioxidant properties in vitro.

Bioactivity-guided isolation is an approach widely used to identify active compounds [43]. We employed this approach to isolate and purify compounds with cholinesterase inhibitory activity from the chloroform fraction by chromatographic methods. This led to the isolation of aknadinine (1), aknadilactam (2), aknadicine (3) and stephisoferuline (4) (Fig. 1). The identification of the compounds was accomplished by analysis of their ^1^H NMR and ^13^C NMR spectroscopic data (Fig. S1-S8, Tables 1, 2 and 3) and a comparison with the published data [21–25]. All of the compounds were found to be hasubanan alkaloids. Although the compounds were first isolated from this plant and from different species of *Stephania* [44], their biological activity was largely unknown. To the best of our knowledge, there was no known information on the effects of hasubanan alkaloids on cholinesterase and oxidative stress.

Inhibitors of cholinesterase are considered the primary treatment for AD. To determine the cholinesterase inhibitory potential of the hasubanan alkaloids, we evaluated them against both acetylcholinesterase and butyrylcholinesterase enzymes. All four compounds exhibited strong inhibitory effects on both acetylcholinesterase and butyrylcholinesterase, and the inhibitory effects were dose dependent (Fig. 2). These compounds inhibited butyrylcholinesterase 2-3.6 times more than acetylcholinesterase. Aknadinine showed the most potent activity against acetylcholinesterase, with an IC_50_ value of 9.36 ± 1.00 µg/mL, while it inhibited butyrylcholinesterase, with an IC_50_ of 4.50 ± 0.53 µg/mL (Table 4). Similarly, stephisoferuline showed the most potent activity against butyrylcholinesterase, with an IC_50_ of 3.97 ± 0.38 µg/mL, and it inhibited acetylcholinesterase, with an IC_50_ of 14.89 ± 1.24 µg/mL. Aknadicine and aknadilactam showed appreciable inhibitory effects on both enzymes. These results revealed the strong potential of the hasubanan alkaloids in the inhibition of acetylcholinesterase and butyrylcholinesterase. Although several classes of alkaloids and N-containing flavonoids have been reported to exhibit cholinesterase inhibitory activity [45, 46], this is the first report of this activity of hasubanan alkaloids.

Anti-cholinesterase drugs inhibit acetylcholinesterase and butyrylcholinesterase through different mechanisms. To understand the mode of enzyme inhibition by the hasubanan alkaloids, we examined the activities of the isolated compounds against acetylcholinesterase and butyrylcholinesterase by a Lineweaver‒Burk plot. From the analysis of the plot (Fig. 3, Table S2), it was found that all of the compounds are uncompetitive inhibitors of acetylcholinesterase and competitive inhibitors of butyrylcholinesterase. These results indicate that these alkaloids inhibit the activity of acetylcholinesterase by binding to a nonactive site of the enzyme or enzyme substrate complex. The mechanism of enzyme inhibition by hasubanan alkaloids is similar to that of isoquinoline, huperazine and indole alkaloids [47].

Molecular docking is an important bioinformatics approach to study interactions between enzymes and ligands. We performed molecular docking experiments to explore the possible interactions between the isolated hasubanan alkaloids and the cholinesterase enzymes acetylcholinesterase and butyrylcholinesterase. The active site of AChE is located at the base of a deep aromatic gorge and consists of multiple subsites, including the catalytic triad (Ser203, His447, and Glu334), the catalytic anionic site (Trp86, Tyr133, Tyr337, and Phe338), the peripheral anionic site (Tyr72, Asp74, Tyr124, Trp286, and Tyr341), the oxyanion hole, and the acyl-binding pocket [48]. In contrast, butyrylcholinesterase (BChE) possesses a structurally similar but compositionally distinct active-site gorge. Its catalytic triad within the CAS is formed by Ser198, Glu325, and His438, which performs the hydrolytic reaction. The PAS of BChE includes residues such as Asp70 and Tyr332, which contribute to ligand binding and regulation of enzymatic activity. Additionally, Trp82 serves as a key residue associated with the choline-binding region in BChE [49, 50]. Among the four alkaloids, aknadinine and aknadilactam showed high binding affinity for acetylcholinesterase followed by aknadicine and stephisoferuline, while aknadilactam and stephisoferuline exhibited the highest binding affinity for butyrylcholinesterase followed by aknadinine and aknadicine (Table 5). The docking results (Fig. 4A) revealed that the hasubanan alkaloids bind to acetylcholinesterase at TRP286 and TYR341 through hydrophobic interactions, which is similar to that of donepezil [45]. These results indicated that these residues may be potential sites for interaction with acetylcholinesterase. On the other hand, all the alkaloids exhibited H-bonding at HIS438 of butyrylcholinesterase (Fig. 4B), indicating a similarity in the mechanism of enzyme inhibition [14]. The differences in the interactions of the compounds with butyrylcholinesterase and acetylcholinesterase may account for their differences in binding affinity of the compounds with butyrylcholinesterase and acetylcholinesterase. The little differences in molecular docking and in vitro results are not surprising, because in the process of docking all the possible spatial structures are taken into account which may not exist stably in biological system. However, the strong inhibition of acetylcholinesterase and butyrylcholinesterase by the hasubanan alkaloids in vitro is supported by the molecular docking studies.

Because oxidative stress takes place with the progression of AD, antioxidants may have the potential to reduce oxidative stress and associated neuropathology [10]. Medicinal plants are an important source of phytochemicals with potential antioxidant activity [51]. Antioxidants act via different mechanisms, such as scavenging free radicals, reducing activity and breaking the peroxide radical chain. We used several assays to investigate the antioxidant activity of the isolated hasubanan alkaloids. DPPH scavenging assays, which are widely accepted as classical models, revealed that these alkaloids have potent antiradical activity (Fig. 5A, Table 6). Hydroxyl free radicals are the most deleterious free radicals formed within the cell and can damage all cellular constituents. All of the compounds were able to scavenge hydroxyl radicals, although to varying degrees (Fig. 5B). Reducing power and total antioxidant activity are indicators of the antioxidant activity of a compound. All of the compounds showed strong reducing power and total antioxidant activity, suggesting their ability to donate electrons or hydrogen (Fig. 5C, D). The peroxidation of lipids results from the oxidation of lipids by free radicals. The antioxidative potential of the hasubanan alkaloids was further confirmed by their ability to inhibit lipid peroxidation in a cell-free system (Fig. 6). Phenolics are well known as natural antioxidants due to the presence of a hydroxyl group in an aromatic ring [52]. The antioxidant activity of hasubanan alkaloids was found to be stronger than that of phenolics and flavonoids from other plants [26, 29].

Alkaloids are a major class of natural compounds many of which exhibit cholinesterase inhibitory activity. The currently used AD drugs, such as donepezil, rivastigmine and galatamine, are all alkaloids. The importance of alkaloids is due to the presence of N on the heterocyclic ring. Physostigmine, the first alkaloid isolated from *Physostigma venenosum*, is a cholinesterase inhibitor that was later modified to the synthetic analog rivastigmine and was approved by the FDA for clinical use in 1996 [45]. Another alkaloid, galantamine, was isolated from *Galanthus nivalis* and approved in 2001. A large number of alkaloids of different classes that exhibit potent cholinesterase inhibitory activities have been isolated from plants. Isoquinoline-type alkaloids are from Amaryllidaceae (*Galanthus* species), indole alkaloids from Apocynaceae, steroidal alkaloids from Buxaceae (*Buxus* species), and quinolizidine-type alkaloids from Lycopodiaceae (*Huperzia* species) [53]. These natural alkaloids have also been used in medicinal chemistry as the starting material for the synthesis of more potent derivatives. Hasubanan alkaloids are a group of alkaloids that differ from morphinan alkaloids because of their five-membered ring [54]. These alkaloids have been found in Stephania species. Although hasubanan alkaloids have been reported to have anti-neuroinflammatory, antinociceptive, antimicrobial and cytotoxic activities [25, 44], their cholinesterase inhibitory potential had not been examined. In this study, we report that hasubanan alkaloids have potential cholinesterase inhibition activity and thus represent a new class of anticholinesterase alkaloids. The strong antioxidant activity detected in this study along with the published anti-neuroinflammatory activity, make hasubanan alkaloids interesting candidates for the treatment of AD. Further studies to address the neuroprotective activity, ability to cross the blood–brain barrier and toxicity of hasubanan alkaloids in mice are warranted.

## Conclusion

In summary, four hasubanan alkaloids, aknadinine, aknadicine, aknadilactam and stephisoferuline, were isolated from *Stephania japonica* that exhibited cholinesterase inhibitory activity in vitro. Molecular docking analysis supported the interaction of hasubanan alkaloids with acetylcholinesterase and butyrylcholinesterase enzymes. The isolated alkaloids exhibited uncompetitive inhibition against acetylcholinesterase and competitive inhibition against butyrylcholinesterase. To the best of our knowledge, this is the first report of hasubanan alkaloids as a new class of cholinesterase inhibitors. All the four alkaloids have potent antioxidant activity and are able to reduce lipid peroxidation. The cholinesterase inhibitory and antioxidant activities of these compounds suggest that hasubanan alkaloids may represent new candidates for the prevention or treatment of AD.

## Supplementary Information

Supplementary Material 1: The following are available online, Figure S1: ^1^H NMR (CDCl_3_, 400 MHz) data of compound 1 (Aknadinine); Figure S2: ^13^C NMR (CDCl_3_, 100 MHz) data of compound 1 (Aknadinine); Figure S3: ^1^H NMR (CDCl_3_, 400 MHz) data of compound 2 (Aknadilactam); Figure S4: ^13^C NMR (CDCl_3_, 100 MHz) data of compound 2 (Aknadilactam); Figure S5: ^1^H NMR (CDCl_3_, 400 MHz) data of compound 3 (Aknadicine); Figure S6: ^13^C NMR (CDCl_3_, 100 MHz) data of compound 3 (Aknadicine); Figure S7: ^1^H NMR (CDCl_3_, 400 MHz) data of compound 4 (Stephisoferuline); Figure S8: ^13^C NMR (CDCl_3_, 100 MHz) data of compound 4 (Stephisoferuline); Table S1: The Grid box set for protein 4AQD and 4BDT to define the binding site for ligand docking; Table S2: Calculation of Km and Vmax values for compounds 1-4 against acetylcholinesterase (AChE) and butyrylcholinesterase (BChE).

## Data Availability

The dataset presented in the study is available from the corresponding author upon request.

## Abbreviations

*S. japonica*: *Stephania japonica*
AD: Alzheimer’s disease
AChE: Acetylcholinesterase
BChE: Butyrylcholinesterase
DTNB: 5,5′-dithio-bis-(2-nitro) benzoic acid
CHF: Chloroform fraction
TLC: Thin layer chromatography
OH: Hydroxyl
2D: Two dimensional
3D: Three dimensional
DPPH: 2,2-diphenyl-1-picrylhydrazyl
IC_50_: Half maximal inhibitory concentration
Aβ: Amyloid beta
MCF: Mixed column fraction
FRP: Ferric reducing power
TBA: Thiobarbituric acid
NMR: Nuclear magnetic resonance
