# Supplementary material for: Isolation and identification of hasubanan alkaloids having anti-cholinesterase and antioxidant activity from the stem Stephania japonica
Source: BMC Complement Med Ther. 2026 Jun 29;26:270. doi: 10.1186/s12906-026-05447-7 (PMC13573421; doi:10.1186/s12906-026-05447-7)
Supplement: Supplementary file 1 [file 12906_2026_5447_MOESM1_ESM.docx]

**Supporting Information**

**“Isolation and Identification of Hasubanan Alkaloids having Anti-cholinesterase and Antioxidant Activity from the Stem *Stephania japonica*”**

**Anik Kumar Dey^1,2^, Md. Yusuf Al-Amin^1,3^, Rafia Ferdous^1^, AHM Khurshid Alam^1^, Aziz Abdur Rahman^1^, Md. Bayazid Hossen^4,6^, Md. Nurul Haque Mollah^4^, and Md. Golam Sadik^1*^**

^1^Department of Pharmacy, University of Rajshahi, Rajshahi-6205, Bangladesh; [anikk.dey18@gmail.com](mailto:anikk.dey18@gmail.com) (A.K.D.); [yusufrupharma@yahoo.com](mailto:yusufrupharma@yahoo.com) (M.Y.A.); [rafiaru22@gmail.com](mailto:rafiaru22@gmail.com) (R.F.); [khurshid.jaist@gmail.com](mailto:khurshid.jaist@gmail.com) (A.H.M.K.A.); [aziz2002@ru.ac.bd](mailto:aziz2002@ru.ac.bd) (A.A. R.); gsadik2@ru.ac.bd (M.G.S.)

^2^ Department of Pharmacy, Gono Bishwabidyalay, Savar, Dhaka-1344, Bangladesh.

^3^Purdue University Interdisciplinary Life Sciences Graduate Program, Purdue University, West Lafayette, IN 47907, USA.

^4^ Bioinformatics Lab, Department of Statistics, University of Rajshahi, Rajshahi-6205, Bangladesh; [bayazid.stat@bau.edu.bd](mailto:bayazid.stat@bau.edu.bd) (B.H.); [mollah.stat.bio@ru.ac.bd](mailto:mollah.stat.bio@ru.ac.bd) (M.N.H.M.)

^5^Department of Agricultural and Applied Statistics, Bangladesh Agricultural University, Mymensingh-2202, Bangladesh

***Corresponding:**  Dr. Md. Golam Sadik, Professor, Department of Pharmacy, University of Rajshahi, Rajshahi-6250, Bangladesh, E-mail: [gsadik2@ru.ac.bd](mailto:gsadik2@ru.ac.bd)

**Figure S1:** ^1^H NMR (CDCl_3_, 400 MHz) data of compound **1** (Aknadinine)

**Figure S2:** ^13^C NMR (CDCl_3_, 100 MHz) data of compound **1** (Aknadinine)

**Figure S3:** ^1^H NMR (CDCl_3_, 400 MHz) data of compound **2** (Aknadilactam)

**Figure S4:** ^13^C NMR (CDCl_3_, 100 MHz) data of compound **2** (Aknadilactam)

**Figure S5:** ^1^H NMR (CDCl_3_, 400 MHz) data of compound **3** (Aknadicine)

**Figure S6:** ^13^C NMR (CDCl_3_, 100 MHz) data of compound **3** (Aknadicine)

**Figure S7:** ^1^H NMR (CDCl_3_, 400 MHz) data of compound **4** (Stephisoferuline)

**Figure S8:** ^13^C NMR (CDCl_3_, 100 MHz) data of compound **4** (Stephisoferuline)

Table S1: The Grid box set for protein 4AQD and 4BDT to define the binding site for ligand docking.

| Protein | Center x | Center y | Center z | Size x | Size y | Size z | exhaustiveness | No. of nodes | energy range |
| --- | --- | --- | --- | --- | --- | --- | --- | --- | --- |
| 4AQD | -0.949 | 2.119 | -13.502 | 106 | 126 | 124 | 8 | 10 | 4 |
| 4BDT | 2.255 | -50.384 | -56.550 | 74 | 86 | 66 | 8 | 10 | 4 |

Table S2: Calculation of Km and Vmax values for compounds **1**-**4** against acetylcholinesterase (AChE) and butyrylcholinesterase (BChE)

| **Sample** | **1/Km** | **Km** | **1/Vmax** | **Vmax** |
| --- | --- | --- | --- | --- |
|  |  |  |  |  |
| **Control** | -1.58 | 0.63 | 9.52 | 0.11 |
| **1** | -1.79 | 0.56 | 12.63 | 0.08 |
| **2** | -2.11 | 0.47 | 17.31 | 0.06 |
| **3** | -2.28 | 0.44 | 18.68 | 0.05 |
| **4** | -1.96 | 0.51 | 12.17 | 0.08 |

| **Sample** | **1/Km** | **Km** | **1/Vmax** | **Vmax** |
| --- | --- | --- | --- | --- |
| **Control** | -0.92 | 1.09 | 2.36 | 0.42 |
| **1** | -0.26 | 3.79 | 1.50 | 0.67 |
| **2** | -0.18 | 5.60 | 0.92 | 1.09 |
| **3** | -0.39 | 2.54 | 1.64 | 0.61 |
| **4** | -0.42 | 2.37 | 1.59 | 0.63 |
